# Gastrodin ameliorates neuroinflammation in Alzheimer’s disease mice by inhibiting NF-κB signaling activation via PPARγ stimulation

**DOI:** 10.18632/aging.205831

**Published:** 2024-05-15

**Authors:** Haoyuan Yin, Renjie Liu, Li Bie

**Affiliations:** 1 Department of Neurovascular Surgery, Bethune First Hospital, Jilin University, Changchun 130021, Jilin, China; 2 Department of Neurovascular Surgery, The First Hospital of Jilin University, Changchun 130021, Jilin, China; 3 Department of Neurosurgery, The First Hospital of Jilin University, Changchun 130021, Jilin, China

**Keywords:** gastrodin, Alzheimer’s disease, PPARγ, neuroinflammation

## Abstract

Aim: We investigated the effects and targets of gastrodin (GAS) for improving cognitive ability in Alzheimer’s disease (AD).

Methods: The targets and mechanisms of GAS were analyzed by network pharmacology. Morris water and eight-arm radial mazes were used to detect the behaviors of 7-months-old APP/PS1 mice. The levels of IBA-1 and PPARγ were examined by histochemical staining, nerve cells were detected by Nissl staining, inflammatory cytokines were measured by ELISA, and protein expressions were monitored by Western blotting. The neurobehavioral effects of GAS on mice were detected after siRNA silencing of PPARγ. Microglia were cultured *in vitro* and Aβ1-42 was used to simulate the pathology of AD. After treatment with GAS, the levels of inflammatory cytokines and proteins were assayed.

Results: Network pharmacological analysis revealed that PPARγ was the action target of GAS. By stimulating PPARγ, GAS inhibited NF-κB signaling activation and decreased neuroinflammation and microglial activation, thereby ameliorating the cognitive ability of AD mice. After silencing PPARγ, GAS could not further improve such cognitive ability. Cellular-level results demonstrated that GAS inhibited microglial injury, reduced tissue inflammation, and activated PPARγ.

Conclusions: GAS can regulate microglia-mediated inflammatory response by stimulating PPARγ and inhibiting NF-κB activation, representing a mechanism whereby it improves the cognitive behavior of AD.

## INTRODUCTION

Alzheimer’s disease (AD) is a cerebral neurodegenerative disease with memory loss and cognitive decline as the main clinical symptoms [1]. Currently, the etiology of AD remains unclear and there are no effective measures for its clinical prevention and treatment [2]. PPARγ, a ligand-activated transcription factor, plays an important role in the pathological process of AD, which can serve as a neuroprotective target [3]. Meanwhile, many PPARγ agonists have been proven to produce neuroprotective effects in both the cellular and animal models of AD [4, 5]. PPARγ is primarily activated by phosphorylation. Research has found that the PPARγ activation by phosphorylation can inhibit the activation of NF-κB [6], thus playing an anti-inflammatory role, while a series of PPARγ activators also exert their actions through phosphorylation [7].

Rhizoma Gastrodiae possesses anti-aging, sedative, hypnotic, anti-inflammatory and anti-oxidant properties. Research has found that it exerts a good effect on cerebral diseases, especially on AD [8]. Gastrodin (GAS), an active component of Rhizoma Gastrodiae, has an inhibitory activity against acetylcholinesterase, which can increase the cerebral blood flow, enhance the brain function, reduce the cerebrovascular resistance and increase the cerebrovascular blood transport; substantial studies have found that GAS has a certain therapeutic effect on AD [9, 10]. Nonetheless, its exact mechanism of action and target have rarely been reported. Through network pharmacological analysis, we found that GAS has a regulatory relationship with a variety of functional proteins, especially with PPARγ. Hence, we further revealed the role and mechanism of GAS-PPARγ in the AD treatment.

## MATERIALS AND METHODS

### Network pharmacological analysis

PharmMapper and SwissTargetPrediction databases were utilized to predict the drug targets, while GeneCards, OMIM and DISGENET databases were used to acquire the disease targets. Venn diagram was drawn with jvenn tool to obtain the intersecting targets between disease and drug. Active component–target network of drug was plotted via Cytoscape 3.8.2, and the interaction network of disease–drug intersection targets was constructed using STRING platform. GO and KEGG pathway enrichment analyses were performed on the disease–drug intersection targets with DAVID database. (1) Acquisition of potential drug targets: the targets with “Norm Fit ≥0.6” in PharmMapper predictions and the targets with “Probability ≥0.1” in SwissTargetPrediction were selected, which were used as the potential targets of GAS after combining and removing the duplications. (2) Acquisition of potential disease targets: “Alzheimer’s disease” was searched in “GeneCards”, “DisGeNET” and “OMIM” databases to obtain the potential disease targets. From the “GeneCards” search results, the targets with “Relevance score ≥ upper quartile” were screened, and from the “DisGeNET” results, the targets with “Score_gda ≥0.1” were screened. After combining them with the targets from other databases and removing the duplications, the potential targets of AD were obtained. (3) Construction of “drug–component–intersection targets” network diagram: By drawing Venn diagram with jvenn tool, the intersecting targets between drug and disease targets were obtained to serve as the potential targets for “drug treatment of AD”. The components and drugs corresponding to intersection targets were screened, with which the “drug–component–target diagram” was constructed via Cytoscape 3.8.2. Targets whose Betweenness Centrality, Closeness Centrality and Degree > medians were selected as the core nodes of the network. (4) Construction of protein–protein interaction (PPI) network diagram: STRING platform was applied to construct PPI network of disease–drug intersection targets. The lowest interaction threshold was set as “highest confidence” >0.4, the isolated nodes were hidden, while the rest was set as default. The data results were imported into Cytoscape 3.8.2 for network analysis, where the nodes represented different targets, and edges represented the relationships between different targets. (5) GO and KEGG enrichment analyses: The disease–drug intersection targets were imported into the online functional annotation tool DAVID, and GO enrichment analysis was conducted from three modules: biological process, cell component and molecular function. Meanwhile, KEGG pathway enrichment analysis was performed. (6) KEGG pathway map drawing: the disease–drug intersection targets were uploaded to the KEGG database, and the KEGG pathway map was constructed with mapper tool.

### Animal experiments

Wild-type C57BL/6J mice and 10-month-old APP/PS1 double-transgenic AD mice were used (Cyagen Biotechnology, Suzhou, China). Mouse experiments were approved by Animal Experimentation Ethics Committee of Jilin University, which conformed to the regulations on animal ethics and welfare.

Normal C57BL/6J mice were defined as Control mice, while APP/PS1 mice were defined as AD mice. GAS was intragastrically administered into the AD mice at 10 mg/kg (GAS-L group) and 20 mg/kg (GAS-H group) once daily. Each of the four groups comprised 10 mice, whose feeding, living and drinking environments were unified. In the mechanism study, we used 10-month-old AD mice and divided them into AD, Si-PPARγ, and GAS+PPARγ groups. The expression of PPAR-γ was inhibited by intracranial injection of siRNA-PPARγ (GenePharma Biotechnology, Shanghai, China) once every three days. In the GAS group, 20 mg/kg GAS was intragastrically administered once daily.

### Morris water maze

The experimental system consisted of a circular pool and an automatic image acquisition and processing system. The pool was 160 cm in diameter, 50 cm in height and 26 cm in depth, where the water temperature was maintained at 21–23°C. We divided the pool into four quadrants, namely 1, 2, 3 and 4, and the platform was located in the middle of the 1st quadrant. One day before the Morris water maze test, the platform was higher than the water surface, and the mice entered the water from the 3rd quadrant. We observed whether the mice could swim to the platform normally, and checked their swimming ability and eyesight. During the first four days of test (place navigation), the mice were placed into water separately from the 2nd, 3rd and 4th quadrants each day to swim freely to the platform. Escape latency referred to the time when the mice reached the platform within 60 s. If they failed to reach the platform within 60 s, they were guided to the platform and stayed for 20 s, and the escape latency was recorded as 60 s. Each quadrant was used once daily at 2 h intervals. After removing the platform, the mice entered the water from the 3rd quadrant, and the number of platform crossings within 60 s by the mice in the area where the original platform was located was recorded for statistical purposes.

### Eight-arm maze

After acclimating to the experimental environment for 1 week, the mice were weighed and fasted for 24 h. Subsequently, normal food was given restrictively following each day of training, with food pellets scattered on various arms and center of the maze. Four mice were then placed in the maze center simultaneously, allowing them to freely feed and explore for 10 min. Repetitive next day’s training proceeded. In this process, the mice were allowed to get familiar with the maze environment without strong stresses. The mice were trained individually: one food pellet was placed in each arm near the outer food container for the mouse to eat freely. After eating the pellet or 10 min later, the mouse was removed and the training of the previous day was repeated twice daily. Four arms were randomly selected, each of which was placed with one food pellet. Various arm doors were closed, and the mouse was placed in the maze center. The arm doors were opened 30 s later, allowing the mouse to move freely and feed in the maze until all the pallets in four arms were eaten. If the food pallets were not finished after 10 min, the experiment would be terminated. Working memory errors, reference memory errors, total number of arms entries and test time were recorded.

### ELISA

The levels of inflammatory cytokines IL-6, IL-1β and TNF-α in the culture medium were assayed. The microglia medium supernatant was detected by ELISA kit (Jiengcheng Bioengineering Institute, Nanjing, China). The OD values were measured at 450 nm, and the results were expressed as pg/mg. The brain tissues were crushed with aseptic scissors, ground with liquid nitrogen, and then lysed on ice with 1.0 ml of RIPA buffer for 30 min. Finally, the supernatant was collected for protein quantification as per the kit instructions.

### Cell experiment

Primary mouse brain microglia (Procell Life Science and Technology, Wuhan, China) were passaged in a tri-gas incubator using special medium (Procell Life Science and Technology, Wuhan, China). Logarithmic microglia were collected and divided into Control, Aβ1-42, GAS-L and GAS-H groups. The Aβ1-42 group was intervened with 25 μM recombinant amyloid 1–42, which was dissolved in PBS and then incubated at 37°C for overnight cross-linking reaction. The GAS groups were pretreated with 10 μM and 20 μM GAS for 6 h, and then treated with 25 μM Aβ1-42.

### CCK-8

Microglia were seeded into 96-well microplates, and incubated in a tri-gas incubator for 4 h after adding each well with 100 μl of phenol red-free medium and 10 μl of CCK-8 reagent (MCE, Shanghai, China). The OD values were measured at 450 nm after changing the medium, and the cytotoxicity was calculated. The results were expressed as %.

### Western blotting

Total proteins were extracted from tissue homogenate and cells with NP-40 lysate, and they quantified by BCA assay. Protein isolation was performed by SDS-PAGE electrophoresis. After 2 h of blockade with 5% skimmed milk powder, the membranes were incubated with TBST-diluted monoclonal primary antibody (Abcam, MA, USA), and further with HRP-conjugated goat anti-rabbit antibody (Abcam, USA). Thereafter, chemiluminescent immunoassay was performed, and OD analysis was accomplished via Image Pro-Plus 6.0.

### Statistical methods

All measurement data were expressed as (± s), and processed using SPSS 17.0. After homogeneity of variance test, two groups of data were compared by two-independent samples *t*-test, while three or more groups of data were analyzed by one-way ANOVA. Subsequent inter-group pairwise comparison was made by LSD method. All of the above tests were two-sided, and the differences were considered statistically significant when *P* < 0.05.

### Data availability statement

The data that support the findings of this study are available from the corresponding author upon reasonable request.

## RESULTS

### Network pharmacological analysis of GAS–AD interaction

**Figure 1:**
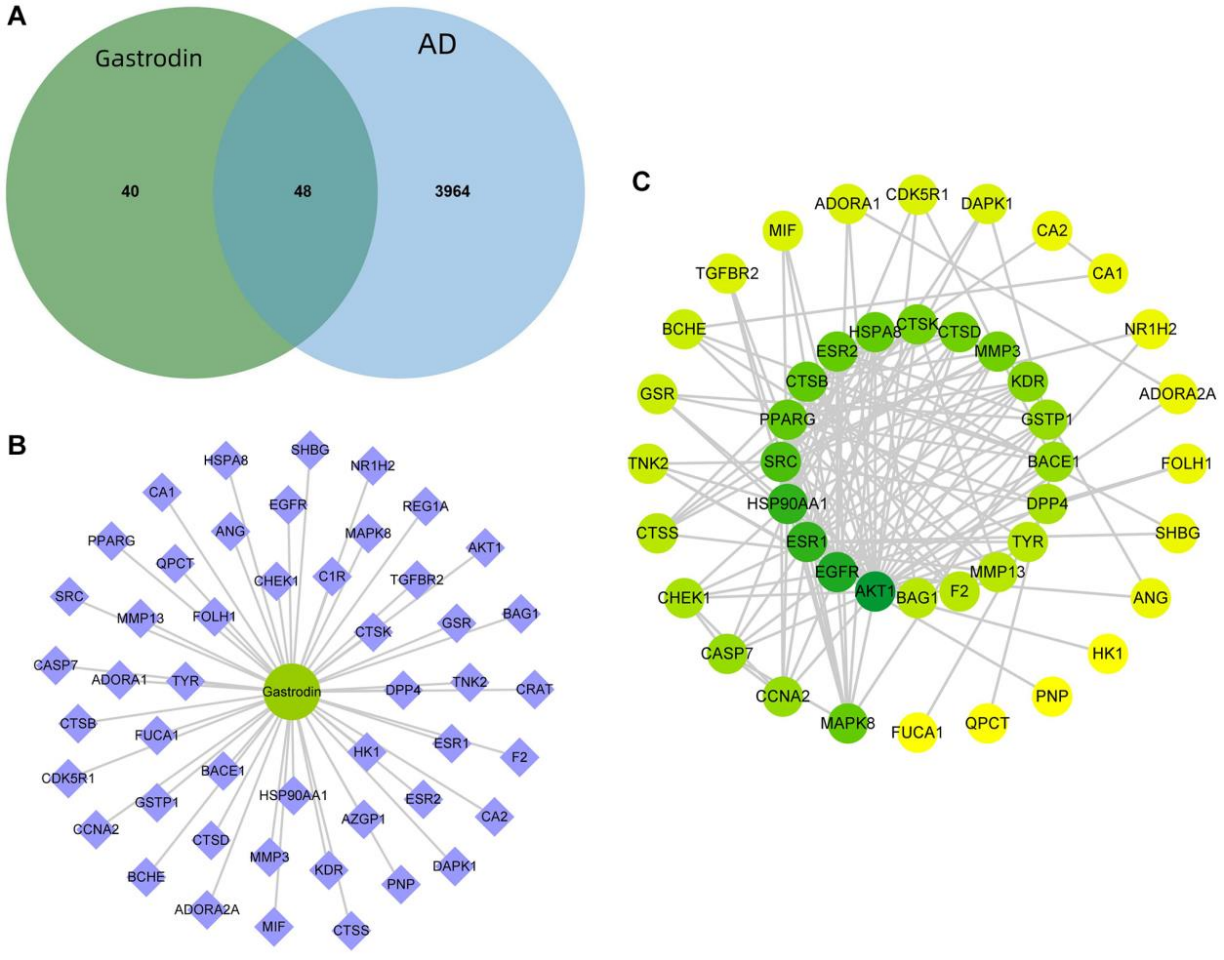
**Prediction of GAS–AD interaction targets.** (**A**) There were 48 GAS–AD intersection targets. (**B**) Drug–component–target network diagram. (**C**) PPI network.

By combining the target prediction results of PharmMapper and SwissTargetPrediction databases, 88 potential drug targets were obtained. Meanwhile, 3,651 targets related to AD were obtained in Genecards database, 673 in DisGeNET database and 183 in OMIM database, which totalled 4,012 after combining them and removing duplications. A total of 48 disease–drug intersection targets were obtained, which were the potential targets for “drug treatment of AD” (Figure 1A). “Drug–component–target network diagram” was constructed, which comprised 1 drug node and 48 disease–drug intersection targets (Figure 1B). Using STRING platform, the PPI network of disease–drug intersection targets was constructed, where there were 44 nodes and 153 edges. After screening the targets according to the network topology attribute values, the top ten ranked by the network degree value were AKT1, EGFR, HSP90AA1, ESR1, SRC, PPARγ, CTSB, ESR2, HSPA8 and CTSD in order (Figure 1C).

**Figure 2:**
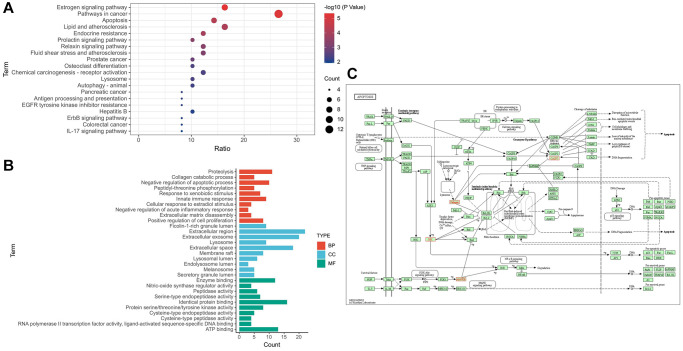
**Target–signal enrichment analysis of GAS–AD interaction.** (**A**) KEGG pathways. (**B**) GO enrichment analysis. (**C**) Results of KEGG enrichment analysis and relevant signal analysis.

Through KEGG enrichment analysis, 55 pathways were obtained, and the top 20 pathways were listed in ascending order of FDR value (Figure 2A). GO enrichment analysis results of 48 disease–drug intersection targets were as follows: 134 biological processes (BP), 52 molecular functions (MF) and 41 cell components (CC). The top 10 BPs, MFs and CCs were displayed separately in ascending order of FDR value (Figure 2B). According to the results of KEGG enrichment analysis, 7 disease–drug intersection targets were enriched in apoptosis signaling pathways, namely CASP7, MAPK8, CTSK, AKT1, CTSD, CTSS and CTSB (Figure 2C).

### Effects and mechanisms of GAS on the AD mice behaviors

**Figure 3:**
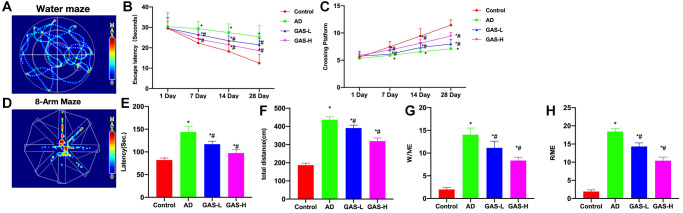
**GAS ameliorated the AD mice behaviors.** (**A**–**C**) Morris (*n* = 10), AD mice exhibited longer escape latency and more platform crossings compared to the Control, while GAS could dose-dependently shorten the escape latency of mice and increase their number of platform crossings. (**D**–**H**) Eight-arm maze (*n* = 10), GAS could shorten the latency, movement distance and open-arms time, showing significant differences from the AD group. ^*^P < 0.05 vs. Control; ^#^*P* < 0.05 vs. AD.

Through the Morris water maze test, we found that the AD mice exhibited longer escape latency and more platform crossings compared to the Control. GAS could shorten the escape latency of mice and increase their number of platform crossings in a dose-dependent manner (Figure 3A–3C). Eight-arm maze results showed that the latency, total movement distance and time spent in open-arms of AD mice were longer than those of Control, while GAS could shorten the latency, movement distance and open-arms time, showing significant differences from the AD group (Figure 3D–3H).

**Figure 4:**
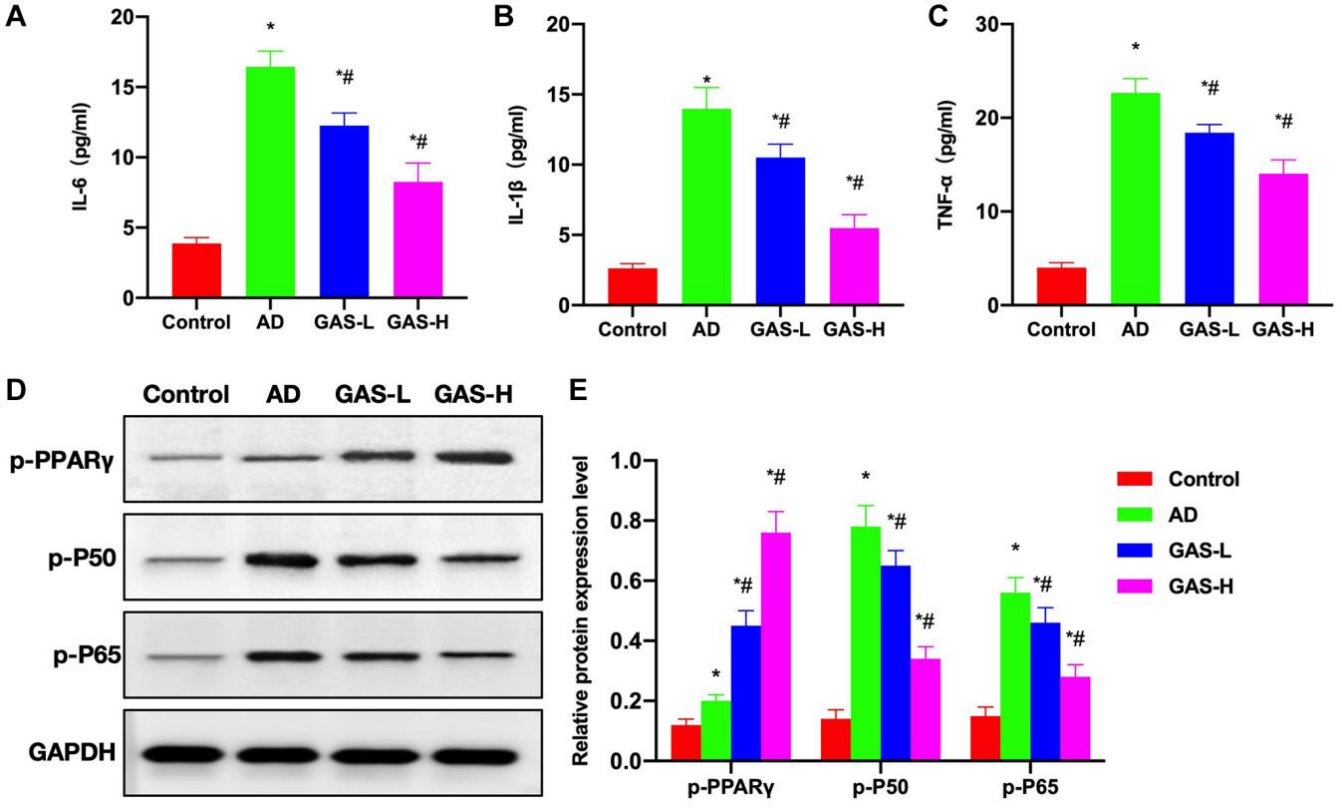
**Effects of GAS on the AD mice pathology.** (**A**–**C**) ELISA (*n* = 10), upregulations of inflammatory cytokines were noted in AD mice, which were significantly higher than those in Control, while GAS could dose-dependently reduce these cytokine expressions. (**D**, **E**) Relative protein expressions (*n* = 5), in the AD group, PPARγ activation was inhibited, p-PPARγ was downregulated, while NF-κB was activated. GAS could activate PPARγ to inhibit the NF-κB signaling and reduce the expressions of p-P50 and p-P65. ^*^P < 0.05 vs. Control; ^#^*P* < 0.05 vs. AD.

According to ELISA assay results, the expressions of inflammatory cytokines were upregulated in AD mice, which were significantly higher than those in Control mice, while GAS could reduce these cytokine expressions in a dose-dependent manner (Figure 4A–4C). Protein assays revealed that PPARγ activation was inhibited in AD group, p-PPARγ was downregulated, while NF-κB was activated. GAS could activate PPARγ, thereby inhibiting the NF-κB signaling and reducing the expressions of p-P50 and p-P65 (Figure 4D, 4E).

### GAS ameliorated the AD mice neurobehaviors by activating PPARγ

**Figure 5:**
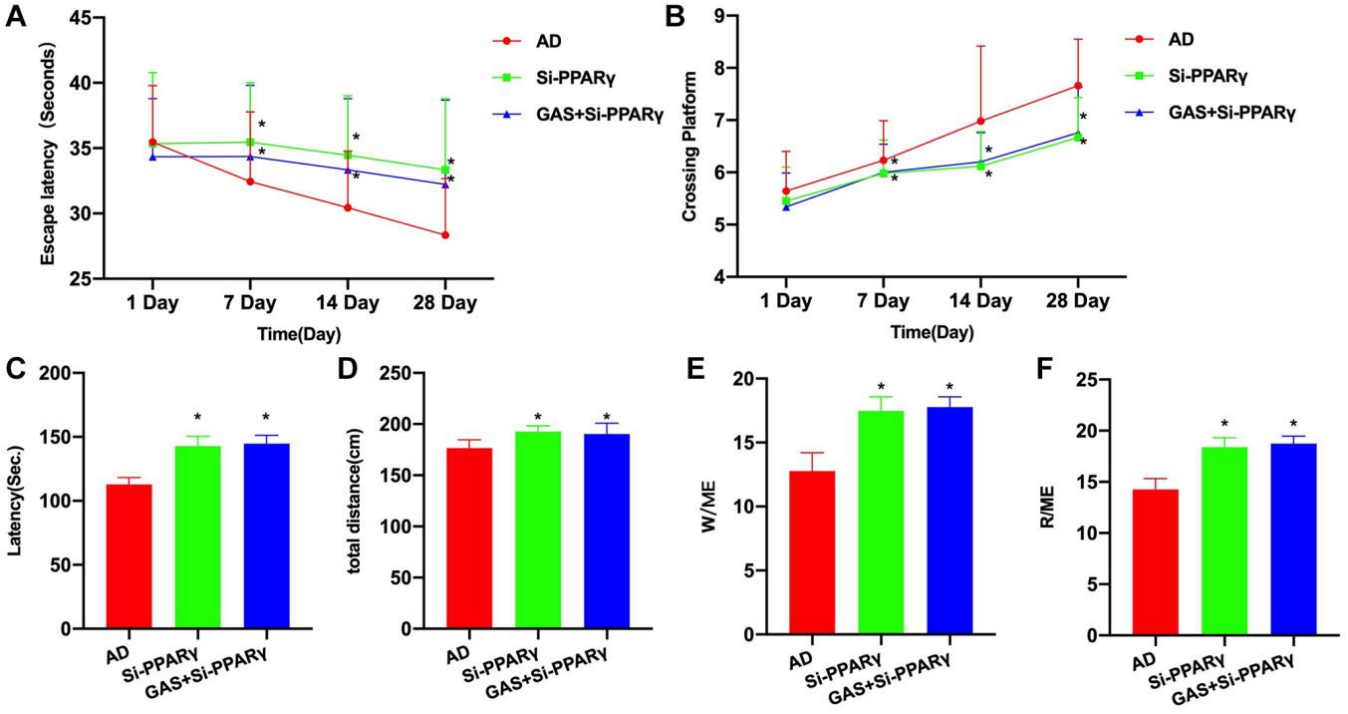
**PPARγ silencing could antagonize the effect of GAS.** (**A**, **B**) Morris (*n* = 10), Si-PPARγ group exhibited longer escape latency and more platform crossings compared to the AD group, while GAS+Si-PPARγ could not improve the mouse behaviors, showing insignificant differences from the Si-PPARγ group. (**C**–**F**) Eight-arm maze (*n* = 10), the latency, total movement distance and open-arms time were all longer in the Si-PPARγ and GAS+Si-PPARγ groups than in the AD group, albeit absence of inter-group differences. ^*^P < 0.05 vs. AD.

After siRNA silencing of PPARγ, the progression of AD was promoted to a certain extent. Morris water maze test revealed that the Si-PPARγ group exhibited longer escape latency and more platform crossings compared to the AD group, while GAS+Si-PPARγ could not improve the mouse behaviors, showing insignificant differences from the Si-PPARγ group (Figure 5A, 5B). According also to the eight-arm maze results, the latency period, total movement distance and open-arms time were all longer in the Si-PPARγ and GAS+Si-PPARγ groups than in the AD group, albeit absence of inter-group differences (Figure 5C–5F).

**Figure 6:**
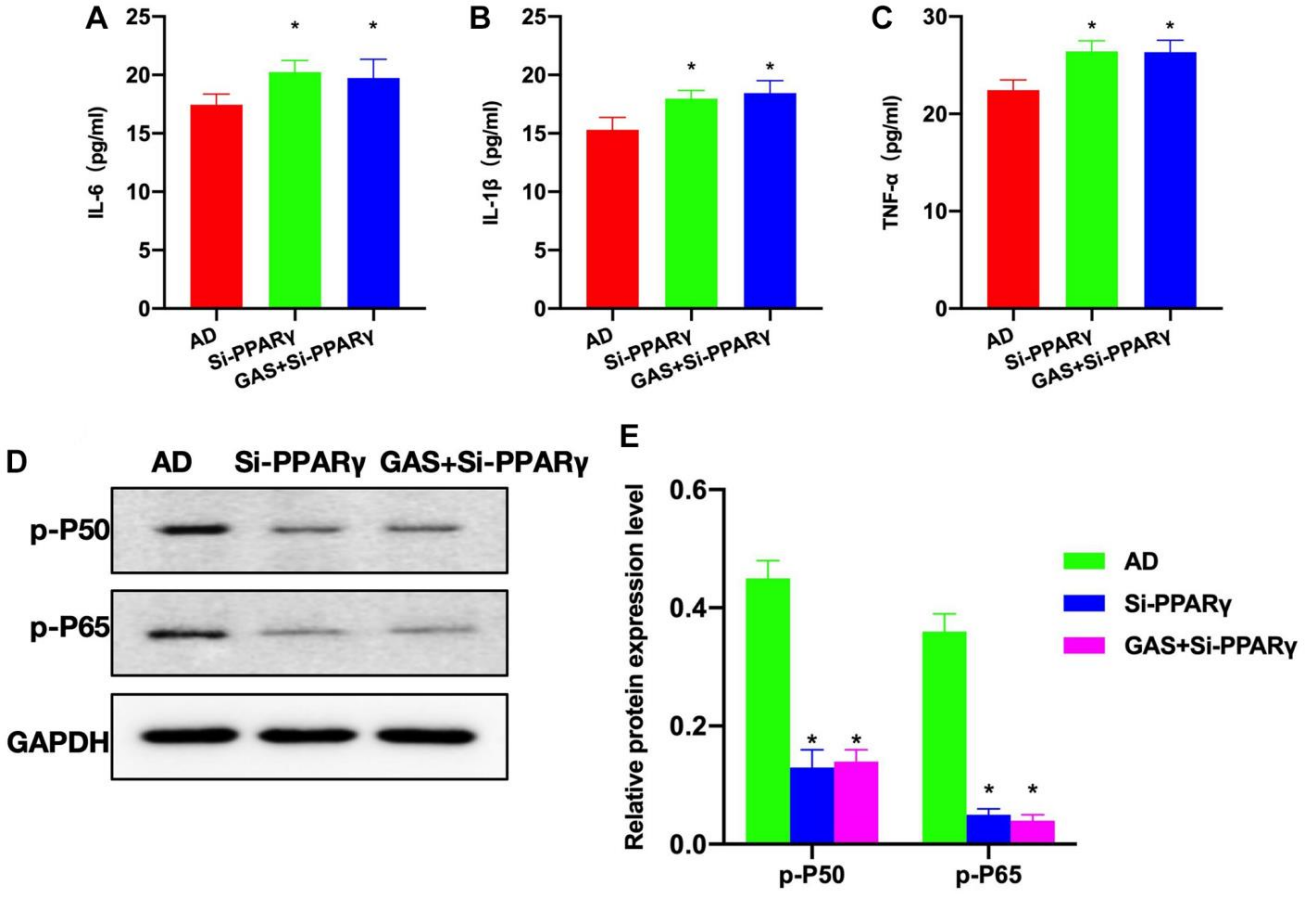
**Effects of PPARγ silencing on the AD mice pathology.** (**A**–**C**) ELISA (*n* = 10), Si-PAPRγ and GAS+Si-PPARγ groups exhibited higher levels of inflammatory cytokines compared to the AD group. (**D**, **E**) Relative protein expressions (*n* = 5), p-P50 and p-P65 expressions differed insignificantly between the Si-PAPRγ and GAS+Si-PPARγ groups. ^*^P < 0.05 vs. AD.

According to the ELISA results, the levels of inflammatory cytokines were higher in the Si-PAPRγ and GAS+Si-PPARγ groups than in the AD group (Figure 6A–6C). Detection of relative protein expressions found no significant differences in the expressions of p-P50 and p-P65 between the Si-PAPRγ and GAS+Si-PPARγ groups (Figure 6D, 6E).

### GAS inhibited the microglial inflammation

**Figure 7:**
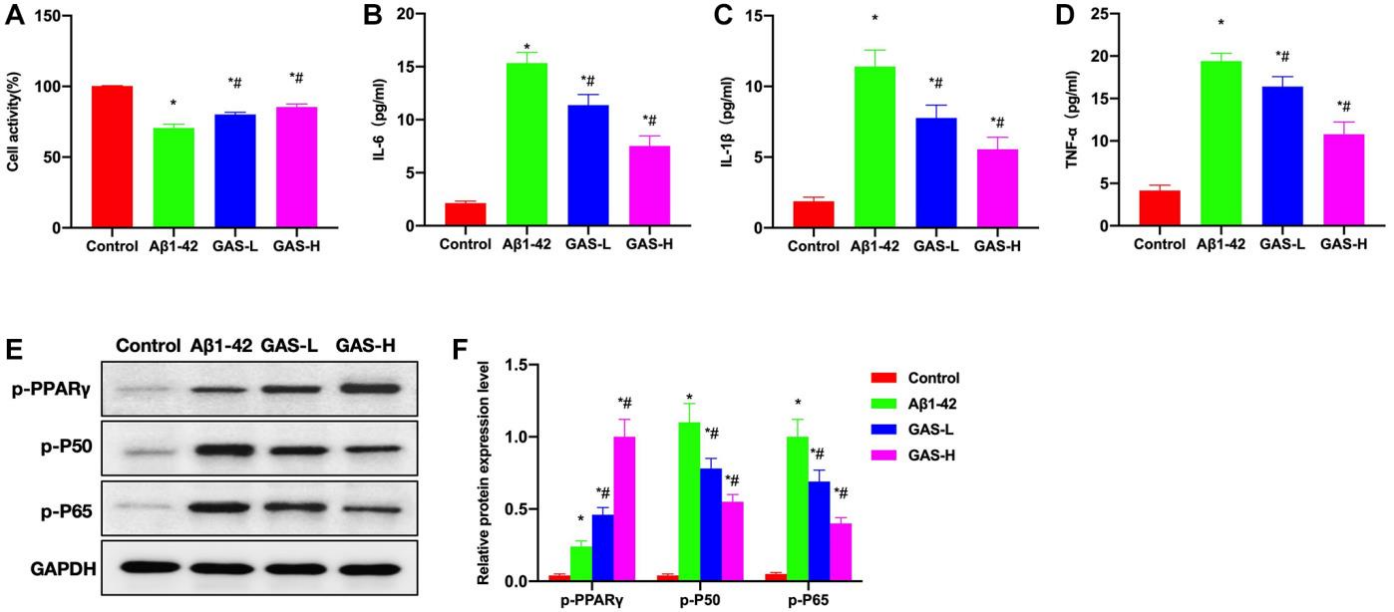
**GAS inhibited the microglial inflammation.** (**A**) CCK-8 (*n* = 3), GAS could inhibit amyloid-induced microglial damage and improve the survival rate. (**B**–**D**) ELISA (*n* = 3), amyloid could induce the expressions of inflammatory cytokines, while GAS could inhibit such changes and lower the cytokine levels in the medium. (**E**–**F**) Relative protein expressions (*n* = 3), GAS could elevate the p-PPARγ level, lower the p-P50 and p-P65 expressions, and inhibit the NF-κB signaling. ^*^P < 0.05 vs. Control; ^#^*P* < 0.05 vs. Aβ1-42.

Aβ1-42 could induce damage and inflammatory response of microglia, and inhibit the activation of PPARγ, while GAS could ameliorate the microglial inflammation and activate PPARγ. Cell viability assay found that GAS could inhibit amyloid-induced damage of microglia and improve the survival rate (Figure 7A). Detection of inflammatory cytokines revealed that amyloid could induce the cytokine expressions, while GAS could inhibit such changes and reduce the cytokine levels in the medium (Figure 7B–7D). Assays of relative protein expressions revealed that GAS could elevate the p-PPARγ level, decrease the expressions of p-P50 and p-P65, and inhibit the NF-κB signaling (Figure 7E, 7F).

## DISCUSSION

Regarding the neuroprotective mechanism of PPARγ pathway in AD, it mainly involves anti-inflammation, anti-oxidation, anti-apoptosis, nerve regeneration promotion and metabolic disorder correction [11, 12]. Research has confirmed that the Aβ deposition-induced inflammatory damage, neuronal apoptosis and oxidative stress are all pathogenic risk factors for AD [13]. Cell experiments have found that PPARγ, as a potential drug target for treating neurodegenerative diseases, chiefly mediates the anti-inflammatory, anti-Aβ, anti-apoptotic and anti-oxidant effects [14, 15]. In terms of anti-inflammation, PPARγ is expressed in both monocytes and macrophages, which, after activation, inhibits monocytes or macrophages from producing inflammatory mediators IL-1β, IL-6, TNF-α, as well as inducible nitric oxide synthase [16]. PPARγ inhibits the proinflammatory gene expressions at the transcriptional level by antagonizing the activities of transcription factors like NF-κB, AP-1 and STAT1 [17], and its agonists can effectively inhibit the inflammatory molecule production mediated by microglia and astrocytes in the central nervous system [18, 19].

GAS, as a small-molecule liposoluble monomer compound extracted from Rhizoma Gastrodiae with good druggability, has recently been approved as a therapeutic drug. Capable of penetrating the blood-brain barrier, it also has great potential in the treatment of neurodegenerative diseases. Previous *in vitro* studies have found that GAS can improve AD pathology by inhibiting Aβ production, suppressing Tau phosphorylation, protecting mitochondria and inhibiting oxidative stress, which has potential to treat AD from multiple mechanisms [20]. In the SAMP8 mouse model, GAS has also been found to improve the cognitive function of mice by inhibiting Aβ production and reducing nerve cell apoptosis [21]. In the SD rat model of Aβ25-35-induced dementia, GAS has also been observed to ameliorate the cognitive function of rats by increasing cholinesterase [22]. Through network pharmacological analysis, we discovered that GAS is correlated with 48 disease–drug intersection targets in AD, and that PPARγ is a potential target of GAS action. According to our overall study, GAS is an activator of PPARγ, which inhibits NF-κB signaling and regulates AD neuroinflammation by promoting p-PPARγ stimulation.

In our study with APP/PS1, a classic mouse model of AD, the 10-month-old mice showed evident cognitive impairment, which was proved by both Morris and eight-arm maze tests. Intervention with GAS could ameliorate the mouse cognitive impairment, and such effect was more pronounced with the prolongation of experiment, and was better at high dose than at low dose. GAS can inhibit the tissue expressions of inflammatory cytokines, whose levels are associated with NF-κB. In AD, NF-κB is abnormally activated. The phosphorylation of P50 and P65 in microglia regulates the level of NF-κB activation, which is also associated with the expressions of inflammatory cytokines. GAS can promote the phosphorylation and stimulation of PPARγ. When PPARγ is stimulated, the activation of NF-κB can be inhibited, leading to reduced phosphorylation of P50 and P65. PPARγ is a regulator in AD neuroinflammation. However, when we silenced it by siRNA, the cognitive impairment progressed in mice, the inflammatory cytokines were upregulated, and GAS failed to inhibit neuroinflammation. Suggestively, PPARγ is the primary target of GAS, and GAS acts through the stimulation of PPARγ. At the cellular level, we found that GAS could inhibit the amyloid-induced microglial damage and inflammatory activation, whose effect was also associated with the activation of PPARγ.

## CONCLUSIONS

Through network pharmacology combined with experimentation, we reveal the anti-AD effects of GAS. Briefly, GAS can regulate the NF-κB signaling activation through PPARγ, thereby inhibiting the progression of neuroinflammation in AD, which has potential as a novel PPARγ inhibitor.
